# Phase transition and dewetting of a 5CB liquid crystal thin film on a topographically patterned substrate†

**DOI:** 10.1039/c9ra02552a

**Published:** 2019-07-12

**Authors:** Palash Dhara, Rabibrata Mukherjee

**Affiliations:** Instability and Soft Patterning Laboratory, Department of Chemical Engineering, Indian Institute of Technology Kharagpur Kharagpur Pin-721302 India rabibrata@che.iitkgp.ac.in

## Abstract

We report thermally induced nematic to isotropic (N–I) phase transition as well as dewetting of 5CB Liquid Crystal (LC) thin films coated on flat and topographically patterned substrates with grating geometry of different line width (*l*_P_) and periodicity (*λ*_P_). On a flat substrate, the nematic to isotropic (N–I) phase transition, which takes place within a temperature range between 31.1 °C and 34.4 °C is fully reversible, with re-appearance of identical Schlieren texture when the sample is cooled down during isotropic to nematic (I–N) transition. Upon further heating beyond N–I transition and annealing at *T* ≈ 65 °C, the film undergoes nucleated dewetting with formation and growth of holes, which eventually merge to form isolated droplets. The morphology of the dewetted structures remains unaltered when the film is cooled to room temperature from this stage, though the features undergo phase transition to the nematic state. In contrast on a topographically patterned substrate, the phase transition cycle is associated with a change of the texture of the film during cooling to the nematic stage. Interestingly the molecules exhibit homeotropic anchoring when *λ*_P_ ≈ 1.5 μm and planar anchoring when *λ*_P_ large (≈10 μm). When heated further to *T* ≈ 65 °C, the film dewets on topographically patterned substrates resulting in a collection of droplets, which are aligned to the substrate patterns when *λ*_P_ is large (≈10 μm). In contrast the dewetted droplets are random and not correlated to the patterns when *λ*_P_ is lower (≈1.5 μm).

## Abstract 2

Thermally induced nematic to isotropic (N–I) phase transition and dewetting of 5CB liquid crystal thin films on flat and topographically patterned substrates.

## Graphical abstract

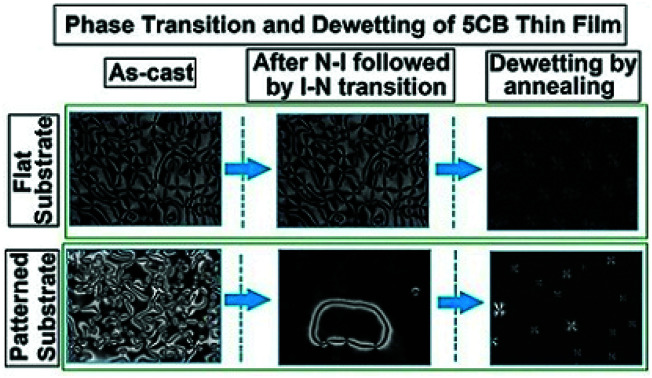

## Introduction

Ultra-thin (thickness < 100 nm) liquid films rupture spontaneously on a non-wetting surface due to amplification of thermally excited surface capillary waves with the formation of random holes that grow with time.^1–7^ Ever since the pioneering contribution by Reiter in 1992,^1^ there has been significant research attention on the spontaneous instability of thin films and dewetting. It is now well established that the spontaneously formed holes grow with time, coalesce, form a network-like structure which eventually disintegrates due to Rayleigh–Taylor instability to form a collection of nearly equal-sized isotropic arrays of droplets, the size (*d*_D_) and periodicity (*λ*_D_) of which scales with the film thickness (*h*_F_). This form of instability is purely hydrodynamic in nature. Thin films of glassy polymers like polystyrene (PS) and polymethylmethacrylate (PMMA) are used as preferred model systems for this form of instability when they are heated above their glass transition temperature (*T*_G_) or exposed to solvent vapor. Switching between liquid and solid states by simple thermal quenching or withdrawing the sample from the solvent chamber allows the possible creation of any intermediate structure and allows their solid state characterization. While such instabilities are undesirable from the standpoint of ultra-thin coatings,^8,9^ instability mediated pattern formation is gaining popularity as a viable non lithographic method for creating meso scale structures spreading over large areas, particularly in conjugation with top down patterning techniques.^10,11^

In addition to polymer thin films, rupture and dewetting of Liquid Crystal (LC) thin films has also been a topic of significant scientific attention.^12–21^ However, the physics associated with dewetting of LC thin films is far more complex, due to the anisotropy in shape of the LC molecules, which results in orientation and positional ordering and is strongly influenced by nature of the interfaces. In case the anchoring or the alignment of the molecules at the film–substrate interface and the film–air interface are different, the mismatch results in texture within the film, which gives rise to an elastic force field within the film. Such elastic effects impart additional resistance against spontaneous rupture of LC films.^14^ Additionally, thermotropic LC films undergo phase transition with increase in temperature, as the orientation and the positional ordering of the molecules change and get distorted.^19^ Phase transition in a thin LC film is known to be a quasi-reversible process near the transition temperature (*T*_C_) as the film returns back to it's original phase once it is cooled below *T*_C_. Reversible phase transition in LC thin films have also been observed when the film is exposed to solvent vapor^22^ or subject to an externally applied electric field.^23^

4-*n*-Pentyl-4′-cyanobiphenyl, C_18_H_19_N (5CB) is a preferred material for performing LC thin film experiments as it remains in the nematic stage at room temperature and undergoes phase transition to the isotropic stage around *T*_C_ ≈ 34 °C. The isotropic stage remains thermally stable till about *T* ≈ 75 °C, after which 5CB starts to evaporate. Interestingly, despite exhaustive literature on dewetting of LC thin films,^12–21^ many aspects of dewetting of a LC thin film is far from being fully understood. While contradicting effects such as pseudo Casimir forces,^17,24^ texture of nematic thin films, elastic stresses arising out of orientation mismatch of the molecules^14^*etc.* are argued to be responsible for dewetting of LC thin films by different groups, it turns out that none of them are scientifically valid as has been clearly documented by Cazabat *et al.*^15^ The genesis of this controversy is attributed to the fact that morphological evolution associated with phase transition in a LC thin film during N–I transition appears very similar to the patterns formed during spinodal dewetting of ultra-thin polymer films.^12,14,17^ However, unlike dewetting, where the patterns evolve with time at a constant temperature, the phase co-existence during N–I transition is driven by thermodynamic equilibrium and the individual domains of the two different phases of different thickness co-exist for hours and evolves only when the temperature is changed. The phase transition in a thin film occurs over a temperature range that is a function of *h*_F_ of the film, and is below the bulk phase transition temperature (*T*_C_). It is fully reversible, as has been shown for 5CB and other liquid crystal thin films by several groups.^15,16^

Careful scrutiny of published literature reveals that there are only few papers that report actual dewetting of LC thin films. Herminghaus *et al.* reported dewetting of 50–80 nm thick 5CB film on silicon substrates, when the film was heated to *T* ≈ 65 °C.^20^ At *T* ≈ 36 °C, which is just above *T*_C,_ holes appeared on film surface, which grew and coalesced to form interconnected droplets with increase in *T*. At *T* ≈ 60 °C, the adjoining threads started to detach from each other, forming isolated droplets. Subsequent cooling showed initiation of rewetting of the substrate at *T* ≈ 48 °C followed by complete filling up of the holes at a temperature close to *T*_C_. Recently, Bandyopadhyay *et al.* reported solvent vapor mediated room temperature N–I phase transition followed by dewetting up to hole formation and hole growth of 5CB films on PS substrates upon prolonged solvent vapor exposure.^22^ The holes gradually healed with the appearance of branched nematic fingers upon subsequent withdrawal of the sample from the solvent chamber.^22^ Few other groups have reported formation of secondary 5CB droplets due to evaporative drying of large 5CB drops, formed during retraction of the contact line.^25,26^ Some research have also been done on the contact line instability of LC drop on solid substrates.^27–30^ Herminghaus *et al.* have also reported instantaneous room temperature dewetting of 5AB_4_ (which undergoes direct phase transition from crystalline phase to isotropic phase at *T* ≈ 10 °C) thin films transferred from an LB trough to a silicon substrate. Clear signatures of both nucleated and spinodal dewetting were observed during the dewetting of the film.^13^

One major limitation of spontaneous dewetting being utilized as a viable patterning technique is the formation of random isotropic structures, which limits their practical applications, despite many advantages such as morphology control by controlling the extent of dewetting and control of feature size by modulating the initial film thickness. This limitation is successfully circumvented in polymer thin films by either pre-patterning the film^31^ or by dewetting the film on a topographically^32–36^ or a chemically patterned substrates,^37–41^ which result in well-ordered patterns spanning over large areas. Of particular interest is dewetting on a topographically patterned substrate, where there is periodic variation in the local thickness of the as cast film along the contours of the substrate patterns. Film rupture is initiated over the top of the substrate features, as the local film thickness is lowest there.^42^ Interestingly, topographically patterned substrates are wildly used in controlling the alignment of LC molecules such as pretilt angle, anchoring energy, multi-display *etc.* within liquid crystal cells, which offer novel in display technologies such as enhanced viewing angle, zenithal bistable displays at reduce power consumption.^43,44^ However, phase transition and dewetting of an LC thin film on a topographically patterned substrates has never been explored before.

In this article we explore thermally induced N–I and I–N transition as well as dewetting of a thin thermotropic 5CB film on a topographically patterned substrate. PMMA substrates comprising of line grating patterns of two different periodicity, *λ*_P_ ≈ 1.5 μm (Type 1 substrate) and *λ*_P_ ≈ 10.0 μm (Type 2 substrate) have been used in our experiments. PMMA coating is extensively used on the walls of LC cells, as 5CB preferentially anchors on it and exhibits planar degenerated anchoring. We also performed phase transition as well as dewetting experiments on a flat PMMA substrate as a control experiment, which clearly allows us to identify the influence of the patterned substrate on both phase transition and dewetting. On a flat PMMA substrate, completely reversible N–I and I–N phase transitions is observed as the system is heated from room temperature to *T* > *T*_C_ and subsequently cooled to *T* < *T*_C_. Further heating of the samples to *T* ≈ 65 °C results in gradual dewetting of the LC films with formation of random dewetted droplets. We observe that the dewetted features transform to nematic state and remain permanent when cooled to room temperature. On the other hand, we show that phase transition on a pattern substrate is no longer reversible, as new texture with very few disclination points appear when the film is cooled below *T*_C_. Further, dewetting of the films on topographically patterned substrates leads to droplets. Interestingly, the dewetted droplets are perfectly aligned on Type 2 substrates only. In contrast, the droplets are random and isotropic on Type 1 substrates, clearly highlighting the importance of commensuration between the *λ*_P_ of patterns and the dominant wavelength of instability of a dewetted 5CB film on a flat surface (*λ*_D_) in achieving ordering.

## Experimental details

Silicon wafer pieces coated with a thick layer of polymethylmethacrylate (PMMA) (*h*_PMMA_ ≈ 500 nm) were used as substrates. The PMMA layer was first obtained by spin coating of PMMA (*M*_w_ ≈ 350k) from a 10% w/v solution in toluene. The PMMA films were vacuum annealed for 12 hours at 60 °C to remove traces of remnant solvent trapped within the entangle chains. For experiments on patterned substrates, some of the PMMA substrates were patterned by pressure assisted capillary force lithography,^45^ using a patterned block of cross linked polydymethylsiloxane (PDMS) as stamp. The PDMS stamp was first exposed to UVO for 30 minute in a UV Ozone chamber (Novascan, USA) before using it for patterning the PMMA film. UVO exposure results in an oxide layer on the surface of the cross linked PDMS stamp, which acts as a diffusion barrier and prevents any possible migration of the uncrossed linked PDMS molecules to the PMMA substrates.^46^ As LC films are extremely sensitive to surface composition, presence of PDMS molecules even in trace amount on the PMMA substrates would alter the anchoring condition and hinders formation of a continuous film. The patterned surfaces had line grating geometry, with two different periodicity (*λ*_P_). The Type 1 patterned substrates had *λ*_P_ ≈ 1.5 μm and the Type 2 substrates had *λ*_P_ ≈ 10.0 μm. Among the Type 1 substrates, gratings with two different line width; *l*_p_ ≈ 0.9 μm (groove width, *λ*_P_ − *l*_p_ ≈ 0.6 μm, Type 1A) and *l*_p_ ≈ 0.4 μm (groove width, *λ*_P_ − *l*_p_ ≈ 1.1 μm, Type 1B) were used in our experiments. The duty ratio (*D*_R_ ≈ (*λ*_P_ − *l*_p_)/*l*_p_) of the Type 1A and Type 1B substrates were 0.66 and 2.75 respectively. The Type 2 patterned substrates had *l*_p_ ≈ 3.0 μm and (*λ*_P_ − *l*_p_) ≈ 7.0 μm (*D*_R_ ≈ 2.33). In all cases, the groove depth was kept constant at *H*_G_ = 120 nm. The morphology of the patterned substrates was characterized with an Atomic Force Microscope (AFM) (Agilent technology, USA, Model 5100) in intermediate contact mode using silicon nitride cantilevers. The corresponding AFM images of the patterned substrates are shown in Fig. S1 of ESI.†

Thin films of 4-*n*-pentyl-4′-cyanobiphenyl (5CB, 99.99% pure Sigma Aldrich, Germany) was spin cast on flat and patterned polymethylmethacrylate (PMMA) substrates from a dilute solution in ethanol. The concentration of the solution was maintained at *C*_n_ = 3.0% to obtain 5CB film with thickness *h*_F_ ≈ 132.1 ± 2.7 nm on flat substrates, which was measured using an imaging ellipsometer (Model: EP3, Accurion GmbH). The drop volume, RPM during spinning and spin durations were maintained at 30 μl, 1200 RPM and 60 seconds respectively, on both flat and patterned substrates to obtain films with same effective film thickness (*h*_E_). After spin coating, the remnant solvent was removed by air annealing the samples in a vacuum desiccator for 6 h at room temperature (*T* ≈ 24 °C). Ethanol was chosen as the solvent, as it did not dissolve or damage the PMMA substrate layer, as was verified from control experiments. The LC coated samples were analysed used an optical microscope (Leica DM 2500) both under bright field and cross polarized light. The disclination point density (|*m*|) was calculated by using image processing software Image J.

Phase transition experiments were performed using a Linkam thermal stage placed on the *X*–*Y*–*Z* stage of the optical microscope, by heating the samples from 24.0 °C to 36.0 °C at a rate of 0.5 °C min^−1^, followed by subsequent cooling back to 24.0 °C. The heating and cooling cycles were performed several times. For dewetting studies the samples were heated to *T* ≈ 65 °C and annealed for different durations of time, in the same stage. The samples were not heated higher temperature as 5CB evaporates around 75 °C. Images were captured under both bright field and cross polarized light.

## Results and discussions

### Phase transition and dewetting on a flat substrate

Proper choice of substrate is essential for obtaining a continuous LC film, as the anchoring of the LC molecules on the substrate strongly influences the formation of the film. It is impossible to obtain a continuous LC film on a substrate that does not exhibit favorable anchoring of the LC molecules. On such surfaces after spin coating, LC starts to retract from the edges forming a large accumulated drop at the center after some time. The reason for using a PMMA substrate is that the polar pendant ester group present in PMMA interacts favorably with the strongly bipolar 5CB molecules, resulting in planar degenerate anchoring and ensuring preferential spreading of a 5CB thin film on a PMMA substrate. Using a PMMA substrate eliminates the possibility of dewetting of the 5CB films due to cohesive interaction between the LC molecules, which is commonly observed on substrates like glass or cross linked PDMS.

**Fig. 1:**
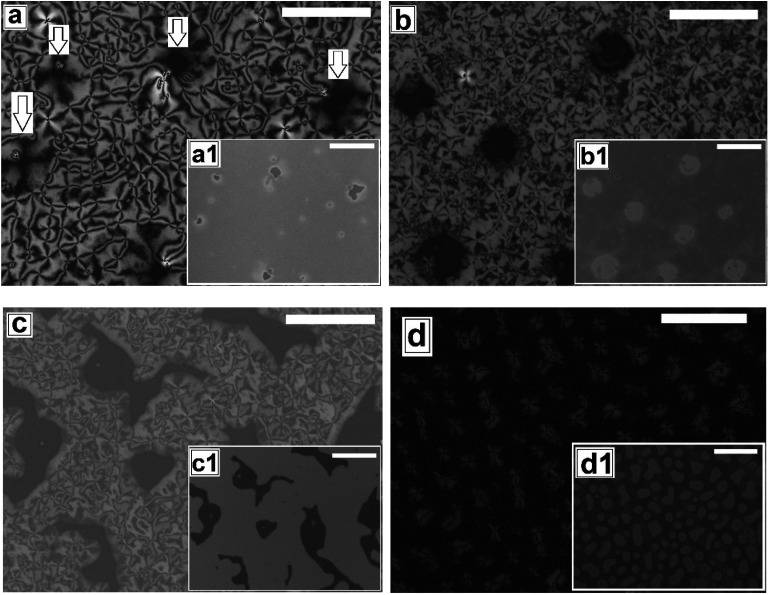
Optical microscope images for the sequence of thermal dewetting of 5CB on flat PMMA. (a) Film rupture and create holes (cross polarized) and inset (bright field) after 30 minutes, (b) holes growth (cross polarized), inset (bright field) after 2 hours and 30 minutes, (c) anisotropic growth of holes in random direction (cross polarized), inset bright field, after 4 hours and 30 minutes (d) isolated random dewetted droplets (cross polarized), inset bright field after 7 hours of annealing. Scale bar 100 μm.

The as cast 5CB film on flat PMMA substrate is flat and exhibits nematic Schlieren texture with densely packed point singularities or disclination points, the density of which is |*m*| ≈ (53 ± )6/(100 μm)^2^. For a 5CB thin film on a PMMA substrate, the anchoring condition is planar degenerate at the film–substrate interface and homeotropic (perpendicular) at the film air interface. The antagonistic anchoring at the two interfaces results in a continuous change in the director field along the depth of the film leading to the formation of the point singularities. The point singularities are lines along which the molecules are oriented perpendicular to the wall. It can be seen in Fig. 1a (as well in ESI Video 1†) that most point singularity has strength *S* = +1 or *S* = −1, where |*S*| = (number of brushes)/4, and is known as the strength of the point singularity.

The phase transition in the 5CB film on a flat PMMA substrate with gradual change in temperature is shown in Video 1 of the ESI.† When the sample is gradually heated from room temperature, phase transition starts around *T* ≈ 30.1 °C, as the sample starts to gradually darken under polarized light. The film transforms fully to the isotropic phase by *T*_C_ ≈ 34.4 °C, as the sample becomes totally dark and remains so upon further heating. Cooling of the sample from this stage leads to re-appearance of the texture at *T* ≈ 33.4 °C, which continues to brighten up till *T* ≈ 29.9 °C. Interestingly, the morphology of the texture as well as the value of |*m*| remains completely unaltered after the film has undergone one complete N–I and I–N phase transition cycle. Subjecting the film to repeated thermal cycle lead to exactly same sequence of events without any change in the film texture or the value of |*m*|.

Next we explore dewetting of a 5CB thin film on a flat substrate, for which the sample was gradually heated to *T* ≈ 65 °C. While the insets in Fig. 1 show the *in situ* bright field image of the films while being annealed at 65 °C, the main frames of Fig. 1 show the microscope images of the same locations 1 hour after cooling the film to room temperature under cross polarized light. It can be seen in the inset (a1) of Fig. 1a that some holes appear on the sample surface after 30 minutes of annealing at *T* ≈ 65 °C. When the sample is cooled to room temperature from this stage, the appearance of the holes remain unaltered under bright field. However nematic texture can be observed at almost all the locations of the holes (marked with arrows in Fig. 1a) under polarized light, which implies that these are not true holes. The contrast observed in the bright field is probably due to variation in the local thickness of the film. Inset of Fig. 1b shows that upon annealing for 2 hours and 30 minutes, the diameter of the holes grow. Further, Fig. 1b confirms that these holes are “true” ruptured holes, as no texture is observed on the base of the holes, when cooled. As *h*_F_ ≈ 132.2 nm, the dewetting observed here is nucleated and therefore the holes appear randomly.^47^ The film may rupture around the defect domains. However, for a LC film, the anchoring exhibited by the molecules at the film–substrate interface is more important. Therefore, we feel that PMMA-5CB interfacial tension has a far insignificant role on dewetting dynamics. It is interesting to note that though the holes are circular in the isotropic state (inset (b1) of Fig. 1b), they become square like when the ruptured LC film is cooled down and transforms to nematic state. Formation of non-circular holes in nematic LC films is attributed to the arrangement of the LC mesogens at the corner of the holes, which has been predicted by Nguyen *et al.* based on molecular dynamics simulations.^21^ With further annealing, the holes grow in random directions (Fig. 1c) and start to coalesce. Finally the growing holes merge and form random isolated droplets (Fig. 1d) after 7 hours of annealing. Once cooled, the droplets in the nematic state exhibit radial texture, though they appear not to be perfectly hemispherical arguably due to non-circular orientation of the LC molecules at the edges in the nematic phase.^21^ The droplets remain stable approximately 12 to 14 hours, after which they start to slowly spread over the substrate, which is attributed to the absorption of the –CN end group of 5CB on polar PMMA.^48,49^ The gradual healing of a fully dewetted 5CB film, is shown in Fig. S2 of ESI.† The film is seen to fully heal in about 2 days.

### Phase transition on patterned substrates

**Fig. 2:**
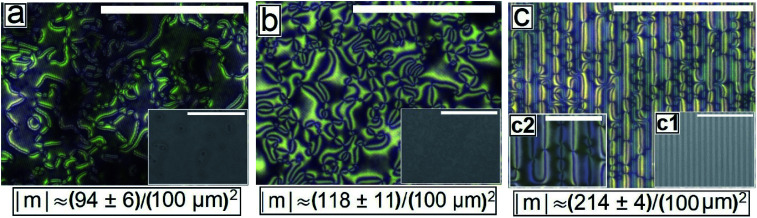
Cross polarized images of as cast Schlieren texture of 5CB continuous film on different PMMA patterned substrates (a) on Type 1A (b) on Type 1B and (c) on Type 2. Insets are bright field images. Scale bar 100 μm. Inset (c2) magnified cross polarized image of the 5CB texture on Type 2 substrate. Scale bar 20 μm.

Fig. 2 shows the as-cast morphology of the 5CB films on Type 1A, Type 1B and Type 2 substrates respectively. Interestingly, the images show the presence of inversion lines on all the patterned substrates which were absent on flat substrates (Fig. 1a). While the inversion lines are random on Type 1 substrates (Fig. 2a and b), they are aligned along the contours of the patterns of Type 2 substrates. The disclination point density, mentioned in each frame of Fig. 2, is seen to depend on the geometry of the substrate. We observe that |*m*| is lower on a Type 1A substrate as compared to that on a Type 1B substrate. This observation provides some idea to correlate |*m*| with the local film thickness on a patterned substrate. When a film is spin coated on a topographically patterned substrate, its top surface is not flat but undulates over the contours of the patterned substrate.^42^ In case the casting solvent preferentially spreads over the substrate, then the undulations are in phase with the substrate patterns (schematically shown in Fig. S1 of ESI†). It is well known that the local film thickness of the film over the substrate stripes is lower than that over the substrate grooves. Based on geometry of substrate patterns and effective film thickness (*h*_E_), a simple calculation is performed (refer to discussion S3 in ESI†) to calculate the local thickness of the film over the substrate grooves (*h*_L1_) and stripes (*h*_L2_). For a film with *h*_E_ ≈ 132 nm, on a Type 1A substrate *h*_L1_ ≈ 204 nm and *h*_L2_ ≈ 84 nm. In contrast, *h*_L1_ ≈ 164 nm and *h*_L2_ ≈ 44 nm on a Type 1B substrate. Recently Ohzono *et al.* reported that the disclination point density decreases with increases in film thickness.^50^ The neighboring disclination points with equal strength and opposite sign tend to merge with each other during the isotropic to nematic phase transition. As a consequence of this coarsening process, number density of the local defect or disclination points reduce. This coarsening process is increasing with the film thickness, so number density of the disclination point decreasing with the increasing film thickness.^50^ In the present case, 5CB was spin coated from a solution with ethanol. It is known that phase transition of 5CB is also occurred due to solvent absorption and desorption from the film.^22^ We argue that at the late stage of spin coating, after deposition of solute (5CB) on the substrates, some residual solvent present within the film. This residual solvent may cause a random orientation of the molecules which impose isotropic state. Further, as the residual solvent evaporates from the film, the molecules organize and come to nematic state. We believe that during this phase transition, coarsening of disclination points may happen. As the coarsening increase with increasing film thickness, the number density of disclination points less at thicker zones and higher at thinner zones. Consequently, more number of point singularities appear on Type 1B substrates, as both *h*_L1_ and *h*_L2_ on this substrate is lower than that on a Type 1A substrate. In contrast to the random inversion lines on Type 1 substrates, Fig. 2c shows that the inversion lines are aligned along the substrate features on Type 2 substrate. It can also be seen in the figure that there are two types of optically distinguishable regions which are present in alternating manner. While one of the regions show long inversion lines with few number of disclination points, the adjacent regions show larger number of disclination points separated by short lines of inversion. Based on the concept that lower local film thickness leads to higher number of disclination points, it can be concluded that the latter regions corresponds to the LC layer over the substrate stripes, where the local film thickness is lower. In contrast, the zones with longer inversion lines and fewer disclination points lie over the substrate grooves, where the local film thickness is higher. Appearance of separate disclination points over both the substrate stripes and grooves is unique and leads to maximization of |*m*| over Type 2 substrates.

**Fig. 3:**
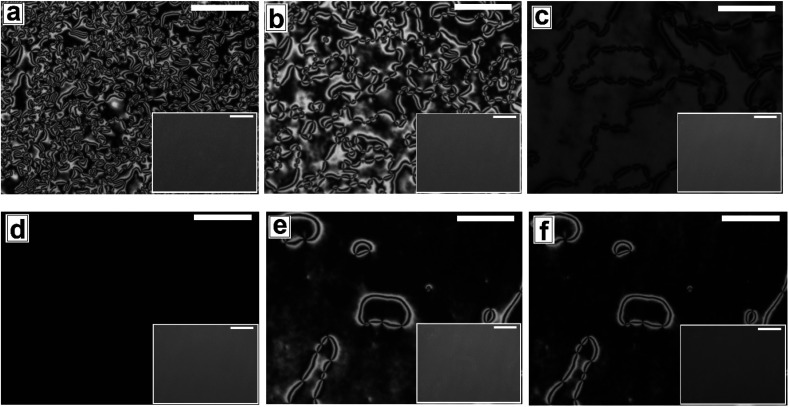
Cross polarized microscope images for nematic to isotropic phase transition of 5CB film on patterned substrates (Type 1B) at (a) 24.7 °C (b) 33.6 °C (c) 35.1 °C (d) 35.5 °C and isotropic to nematic phase transition at (e) 30.6 °C (f) 24.6 °C. Inset corresponding bright field images. Scale bar 100 μm.

Fig. 3 shows the N–I and I–N transformation of a 5CB film with *h*_E_ ≈ 132.2 nm on Type 1B substrates. The corresponding video is attached as Video 2 in the ESI.† For phase transition studies, the samples were heated from 24 °C to 36 °C in step of 0.5 °C min^−1^ and subsequently cooled down. As can be seen in the ESI Video 2,† inversion lines start to disappear when heating starts, resulting in gradual reduction of |*m*| with increase in temperature, which is shown in Fig. 5. The rate of disappearance of the inversion lines increases when temperature reaches ≈33.6 °C. From the video it can be seen that the inversion lines shrink in length and in the process the point singularities at the two ends of each inversion lines are pulled closer, which is followed by merger of the point singularities and complete disappearance of the inversion lines. It has been reported by Nehring and Saupe in the classic paper on Schlieren texture that the tendency of shrinkage and disappearance of inversion lines is possible only in “Inversion lines of the second kind”.^51^ Such inversion lines appear only when the surface molecules which are supposed to be parallel to the surface get tilted out of plane of the layer, which we argue must be happening in the present case due to the presence of the substrate patterns. With progressive heating of the samples, the inversion lines disappear completely and the film turns totally dark at *T* ≈ 35.5 °C (Fig. 3d) under cross polarized light signifying transition to the isotropic state. Nearly identical textural evolution sequence is also observed in films coated on Type 1A substrates, which is shown in ESI Video 3.†Fig. 3e and f show the gradual morphology transition when the sample is cooled down from 36 °C to room temperature. Important to note that unlike that observed on a flat substrate, the texture of the film on the patterned substrates change completely when cooled to the nematic state, with the appearance of very few narrow brushes. The disclination point density drops drastically, to |*m*| ≈ (3 ± 2)/(100 μm)^2^. The overall appearance of film remains dark, which indicates that the molecules remain in a state of homeotropic anchoring even in the nematic phase. The corresponding bright field images confirm that the film remains totally featureless during the entire forward N–I as well as backward I–N phase transition process. Near identical texture is observed on Type 1A substrates as well. The alignment or orientation of liquid crystal molecules on the patterned substrates leads to a very complex situation. We believe that molecules align parallel to the groove direction following the Berreman's model.^52^ The molecules may orient parallel to the side wall of the grooves which is perpendicular to the base. At the same time, the molecular orientation is also parallel on the ridges and groove bottoms. It may create a complex ordering of molecules in the bulk. The elastic energy penalty in an as-cast film on a patterned substrate is high due to the presence of large number of disclination points. Also the inversion walls are associated with higher energy, as they contain local molecular order. Combination of these two effects lead to much higher level of energy trapped in the as cast film on a patterned substrate, as compared to that in a flat LC film. The large disclination point density is probably attributed to the mechanism of film formation by spin coating, where the solute molecules phase separate from the solution layer and deposit inside the substrate grooves and stripe tops independently, and the initial orientation of these molecules are entirely governed by the substrate material. Particularly for the LC molecules that deposit inside a substrate groove, adjacent locations of the base and the side walls of the groove impose different direction of the director of the molecules, resulting in much higher numbers of inversion lines or defects.^44^ As a consequence of that the spin coated as-cast film on patterned substrates entrap large amount of energy at room temperature. During the phase transition, when such a film is heated to the isotropic state, these trapped excess energy within the disclination points and inversion lines get released and the molecules reorganize themselves. We feel that in the isotropic state, the bulk molecules reorganize and form a minimum energy configuration that is imposed by the orientation of the free surface of the film. This is likely to be associated with physical reorganization of the molecules due to Laplace pressure arising out of the curvature of the free surface of the film at the isotropic state. This however could not be confirmed as the films were too sticky to perform any AFM imaging. It should be mentioned that the Laplace pressure at the nematic phase is different than the isotropic phase due to different molecular orientation.^53^ Further, during cooling (I–N), it is observed that the texture of the film does not change but shows new texture with few disclination points. We believe that after achieving minimum energy configuration, the orientation of bulk molecules do not change. It has been reported previously that with decreasing *λ*_P_ of patterned substrates, the molecules orient homeotropically.^54,55^ Here, we also observe similar trend as the film becomes dark on Type 1A and 1B substrates (*λ*_P_ ≈ 1.5 μm) after I–N transition under the crossed polarized microscope which indicates homeotropic alignment (Fig. 3f) of the bulk molecules.

**Fig. 4:**
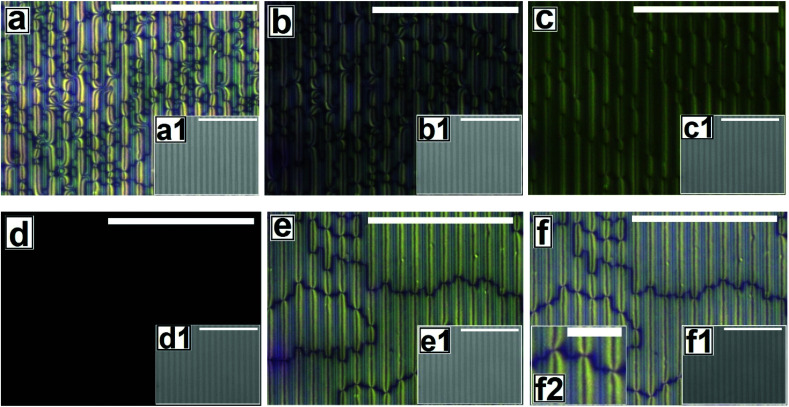
Cross polarized microscope images for nematic to isotropic phase transition of 5CB film on Type 2 patterned substrates at (a) 25.0 °C (b) 33.6 °C (c) 34.8 °C (d) 35.6 °C and isotropic to nematic phase transition at (e) 30.3 °C (f) 25.1 °C. Inset corresponding bright field images. Scale bar 100 μm. Inset (f2) magnify polarized image, scale bar 20 μm.

**Fig. 5:**
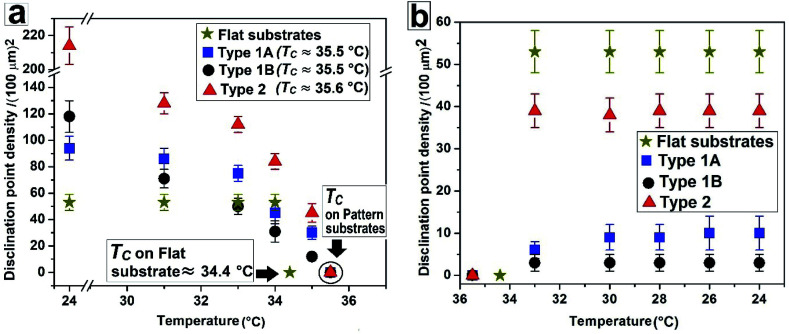
Change of disclination point density |*m*| with temperature during (a) nematic to isotropic and (b) isotropic to nematic phase transition.

Fig. 4 and Video 4 in the ESI† show the gradual N–I and I–N transition of a 5CB film on a Type 2 substrate of same *h*_E_ as used in Fig. 3. Unlike on a Type 1B substrate, where the inversion lines show shrinkage and |*m*| decreases rapidly with heating, in this case, there is hardly any change in |*m*| initially as can be clearly seen by comparing Fig. 4a and b. However, around a temperature of 34.8 °C, the disclination points over the substrate stripes join up and disappear while the inversion lines remain intact over the substrate grooves (Fig. 4c). The above observation clearly highlight that the “inversion line of first kind” over the substrate groove (which do not shrink) and “inversion line of second kind” over the substrate stripes (which shrinks)^51^ co-exit laterally. This happen due to variation in the local thickness of the film within the grooves and stripes as discussed previously. Further, heating of the sample leads to complete darkening of the film indicating transition to isotropic phase (Fig. 4d). The temperature at which the texture completely disappears is 35.6 °C (*T*_C_) which is slightly higher than that observed on Type 1 substrates (*T*_C_ ≈ 35.5 °C) as well as the clearing temperature on a flat substrate (*T*_C_ ≈ 34.4 °C) as can be seen in Fig. 5. This observations allow us to conclude that the clearing temperature increases with increasing the value of |*m*| as the molecules require larger amount of energy to overcome the local order imposed by inversion line when more number of |*m*| are present. Fig. 4e and f show that upon cooling, similar to observed in Fig. 3, a nearly homogeneous film is obtained with very low |*m*| and few inversion lines in the nematic state. However, the film is bright which is in clear contrast to a dark film seen in Fig. 3f. This means that unlike on Type 1 substrates the molecules are no longer homeotropic state in the present case. The difference in the state of anchoring on Type 1 substrates and Type 2 substrates, after the first I–N transition can be explained based on the fact that as the pattern periodicity increases, the degree of ordering of the molecules imposed by the side walls of the grooves decreases. Consequently, the molecules progressively loose the degree of homeotropic ordering over Type 2 substrates over wider grooves. Such transition in molecular ordering due to variation in the periodicity of a topographically patterned substrate has been previously reported by Clark and coworker.^55^ Unfortunately, we did not have any other stamp with intermediate *λ*_P_ to narrow down the feature dimension around which the transition in anchoring occurs.

Fig. 5 summarises the observations related to the gradual change in |*m*| with temperature on different substrates during N–I and I–N transition. It can be seen that |*m*| does not change at all on a flat substrate, during the forward N–I transition, and the film suddenly turns dark with disappearance of all texture within a narrow temperature window between 34 °C and 34.4 °C. In contrast, on all the patterned substrates |*m*| starts to decrease gradually from *T* ≈ 30 °C due to shrinkage of the inversion lines followed by merging of point singularities and their subsequent disappearance around *T*_C_ ≈ 35.5 °C for Type 1 and *T*_C_ ≈ 35.6 °C for Type 2 substrates. All the samples turn totally black at a clearing temperature (*T*_C_). Most importantly, on all the patterned substrates, any subsequent N–I followed by I–N transition by heating and cooling results in exactly identical texture as obtained after cooling the sample for the first time, or the morphology obtained after the first I–N transition. This clearly indicates that the non-equilibrium orientation of the molecules obtained by spin coating of a LC film on a topographically patterned substrates transforms to equilibrium configuration during the first N–I transformation, which subsequently remains unaltered.

**Fig. 6:**
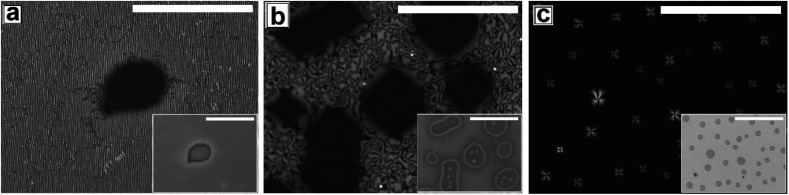
Optical microscope images in cross polarized mode for sequence of thermal dewetting of 5CB film on grating PMMA (Type 1B), by annealing at 65 °C (a) formation of holes after 2 hours, (b) holes growth and merge after 4 hours and (c) randomly oriented droplets after 5 hours and 30 minutes of annealing. Insets are corresponding bright field images. Scale bar 100 μm.

**Fig. 7:**
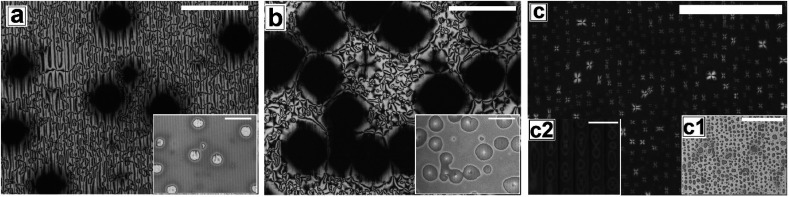
Cross polarized images for morphological evolution of 5CB continuous film by thermal dewetting at 65 °C on Type 2 substrates. Sequence of dewetting (a) holes formation after 2 hours (b) holes growth after 4 hours (c) drop aligned along the pattern direction after 9 hours of annealing. Insets show corresponding bright field images. Scale bar 100 μm. Inset (c2) shows the magnified polarized image and scale bar 20 μm.

In Fig. 6 and 7, we show the dewetting of a 5CB film with *h*_E_ ≈ 132 nm on Type 1B and Type 2 substrates respectively. It can be seen that in both cases the initial rupture of the film is with the formation of random holes, which are not correlated to the substrate patterns. Rupture of a thick polymer film on a patterned substrate with nucleation of random holes has been observed earlier in the context of dewetting of polymer films.^56^ Typically, in such cases the retracting contact line leaves behind tiny droplets along the contours of the substrates patterns. This results in the formation of aligned droplets along the grooves of the patterned substrates, over longer time scales, despite having random initial holes. This mechanism of drop formation is obviously prevailing on a Type 2 substrate, which can be seen in Fig. 7c. In contrast on a Type 1 substrate, the final dewetted morphology comprises of a random collection of large droplets spanning several stripes (Fig. 6c). Each droplet is hemispherical in shape and exhibits radial texture. Much narrower stripes (*λ*_P_ ≈ 1.5 μm) in Type 1 substrates as compared to those on Type 2 substrate (*λ*_P_ ≈ 10 μm) fail to confine the retracting contact line, and the rim of the growing holes retracts similar to that on a flat surface, eventually resulting in larger droplets uncorrelated to the substrate patterns. Thus it becomes evident that for nucleated dewetting of a liquid crystal thin film, there is a threshold *λ*_P_ over which pattern directed dewetting can be achieved.

## Conclusion

In this article, we have reported phase transition and thermal dewetting of 5CB thin film on a flat as well as topographically patterned PMMA substrates. The as-cast film on flat substrate exhibits Schlieren texture with large number of disclination points. As has been widely reported in literature, completely reversible phase transition is observed on a flat surface as the film is first heated for the forward N–I transition and subsequently cooled for the reverse I–N phase transition. The phase transition temperature (*T*_C_) for 5CB film on flat surface is found to be 34.4 °C. Further heating of the film and annealing it at 65 °C leads to nucleated dewetting resulting in random collection droplets.

On a topographically patterned substrate, the Schlieren texture of the as-cast film comprises inversion lines, in addition to disclination points. The precise texture of the film is also seen to depend on the geometry of the substrate. While the inversion lines are randomly oriented on Type 1 substrate (*λ*_P_ ≈ 1.5 μm), they are seen to be aligned along the contours of the substrate patterns on a Type 2 substrate (*λ*_P_ ≈ 10 μm). Further, the disclination point density |*m*| is also seen to depend on pattern geometry. Similar to that observed on a flat substrate, when heated the 5CB film on a topographically patterned substrate undergoes N–I transition. However, upon cooling from the isotropic state the film does not show the initial texture, indicating irreversible phase transition which is a novel and unique observation. Further, while the molecules in the film after cooling are seen to be in the homeotropic phase on Type 1 substrate, they exhibit planar orientation on Type 2 substrate. Further, heating of the film on both type of patterned substrates leads to dewetting of the film. While the dewetted droplets are seen to be aligned along the contours of the substrate pattern on a Type 2 substrate, they are found to be random and non correlated to the substrate patterns on Type 1 substrates. Thus, pattern-directed dewetting of a thin LC film leads to myriad of structures, depending on the geometry and periodicity of the substrate.

## Conflicts of interest

There are no conflicts to declare.

## Supplementary Material

RA-009-C9RA02552A-s001

RA-009-C9RA02552A-s002

RA-009-C9RA02552A-s003

RA-009-C9RA02552A-s004

RA-009-C9RA02552A-s005
