# Supplementary material for: Phase transition and dewetting of a 5CB liquid crystal thin film on a topographically patterned substrate
Source: RSC Adv. 2019 Jul 12;9(38):21685–94. doi: 10.1039/c9ra02552a (PMC9066433; doi:10.1039/c9ra02552a)
Supplement: RA-009-C9RA02552A-s001 [file RA-009-C9RA02552A-s001.pdf]

This journal is © The Royal Society of Chemistry 2019

Phase Transition and Dewetting of 5CB Liquid Crystal Thin Film on Topographically Pattern Substrate

Palash Dhara and Rabibrata Mukherjee*

Instability and Soft Patterning Laboratory, Department of Chemical Engineering, Indian Institute of Technology Kharagpur, Kharagpur, Pin-721302, India.

*e-mail: rabibrata@che.iitkgp.ac.in

Supporting Information

S1: AFM images of the bare patterned PMMA substrates

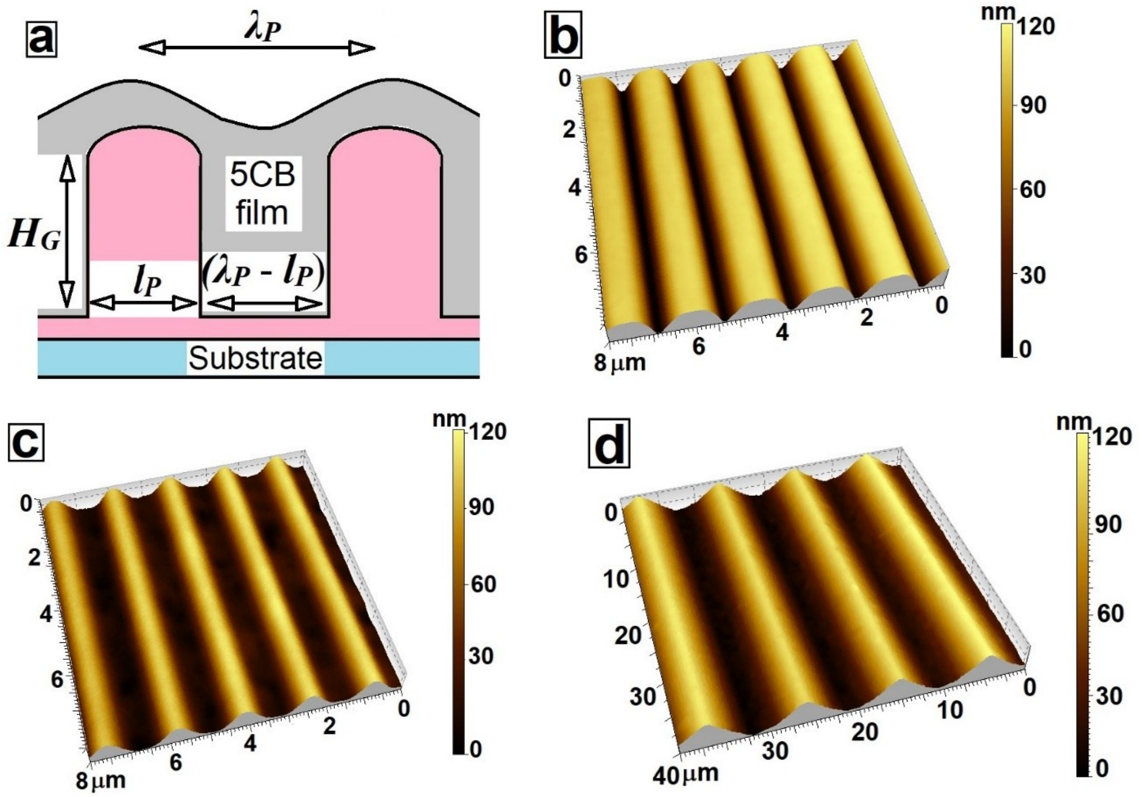

S1: *(a) Schematic of 5CB coated on patterned substrates with different nomenclatures. AFM images of bare PMMA patterned substrates of (b) Type 1A (c) Type 1B and (d) Type 2 substrates.*

S2: Healing of dewetted structures of 5CB on PMMA substrate:

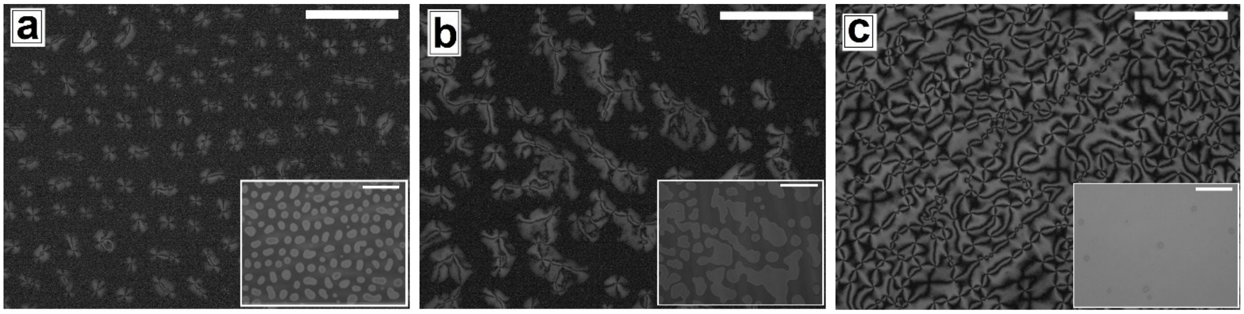

S2: Polarized microscope images of (a) 5CB dewetted droplets on PMMA substrate. Spreading and healing of dewetted droplets at room temperature after (b) 15 hours and (c) 40 hours. Insets are bright field images. Scale bar 100 µm.

S3: Calculation for h_{L1} and h_{L2} on Type 1A and 1B substrates:

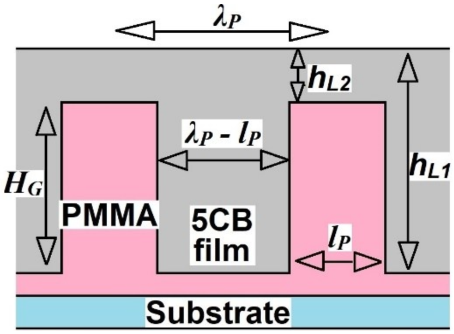

Schematic with different nomenclatures for 5CB thin film coated on patterned substrate.

From the mass balance, we get,

$$\{h_{L1} *(\lambda_P - l_P)\} + (h_{L2} * l_P) = h_E * (\lambda_P) \quad \text{(i)}$$

$$h_{L1} = h_{L2} + H_G \quad \text{(ii)}$$

Where, h_{L1} = Local thickness of the 5CB film on patterns grooves regions.

h_{L2} = Local film thickness on patterns stripes regions.

λ_P = Patterned periodicity.

l_P = Line width.

(λ_P - l_P) = Groove width.

H_G = Groove depth.

h_E = Effective film thickness

Now, for **Type 1A** substrates:

λ_P = 1.5 µm

l_P = 900 nm

$(\lambda_P - l_P)$ = 600 nm

H_G = 120 nm

h_E = 132 nm

$\{h_{L1} *(\lambda_P - l_P)\}+ (h_{L2} * l_P) = h_E * (\lambda_P)$ (i)

$h_{L1} = h_{L2} + H_G$ (ii)

Putting equation (ii) into equation (i), we get,

$\{(h_{L2} + H_G) *(\lambda_P - l_P)\}+ (h_{L2} * l_P) = h_E * (\lambda_P)$ (iii)

Putting all the value,

We get,

h_{L2} = 84 nm & h_{L1} = 204 nm.

Similarly, for **Type 1B** substrates:

λ_P = 1.5 µm

l_P = 400 nm

$(\lambda_P - l_P)$ = 1100 nm

H_G = 120 nm

h_E = 132 nm

Putting the values into equation (i) and (ii),

We get,

h_{L2} = 44 nm & h_{L1} = 164 nm.
